# Exploration of the population size of men who have sex with men in Hainan colleges: A cross-sectional study based on geographical social network App

**DOI:** 10.1371/journal.pone.0358788

**Published:** 2026-09-21

**Authors:** Shi Chen, Xin Chen, Rui Feng, Guihua Chen, Chunyun Lu, Donghui Zhao, Zhaoqian Wang, Dee Yu

**Affiliations:** 1 Key Laboratory of Tropical Translational Medicine of Ministry of Education, School of Public Health, Hainan Academy of Medical Sciences, Hainan Medical University, Haikou, Hainan, China; 2 Hainan Provincial Center for Disease Control and Prevention, Haikou, Hainan, China; Kerman University of Medical Sciences, IRAN, ISLAMIC REPUBLIC OF

## Abstract

Men who have sex with men (MSM) have historically been a high-risk group for sexually transmitted diseases. In Hainan, the incidence of HIV and syphilis among MSM has increased rapidly. Additionally, there is a growing number of young students being diagnosed with HIV, with MSM identified as the primary demographic affected. However, the size and distribution of the MSM population within colleges and universities in Hainan Province remains unknown. In this study, the Blued App was employed to collect data on users within Hainan colleges and universities from May 9 to May 18, 2023. Using the users information and questionnaire survey data, we applied the multiplier method to estimate the population size of student MSM. Multivariate and geographic density analyses integrating survey data were conducted to identify regional disparities. A questionnaire survey revealed that 89.28% of student MSM reported using the Blued App, and 76.00% of the individuals utilized it for seeking sexual partners. The estimated population size of MSM in colleges across Hainan Province was 3504 (95%CI: 3464–3544), with Haikou and Sanya identified as hotspots. The proportion of active users was 33.13% (860/2596). Notably, Danzhou and Wenchang, relatively small urban areas with limited Blued registrants, exhibited high proportion of active users (42.22% and 41.30%). These findings suggest that particular attention should be directed towards sexual health education, and sexually transmitted disease surveillance in specific colleges.

## Introduction

The role of men who have sex with men (MSM) in the sexually transmitted diseases (STD) epidemic is considerable. In China, the national prevalence of HIV among MSM from 2001 to 2018 was estimated to be 5.7% [1]. The prevalence of HIV among student MSM was 4.4% [2]. Additionally, a meta-analysis reported that the HIV prevalence among MSM in high schools and colleges across China was also 3.8% [3]. Moreover, the prevalence of HIV among MSM who had heterosexual sex was significantly higher, at 9% [4]. In the United States, MSM comprised 53% and 67% of new HIV cases in 2006 and 2014, respectively [5,6]. Globally, MSM are experiencing an escalating risk of HIV infection, with the number of cases rising [7,8]. Furthermore, the death rate among MSM is much higher (8%) [9]. The HIV epidemic among Chinese college students has worsened due to technological advancements [10,11].

The utilization of online platforms and social media may elevate the risk of STD by facilitating the search for sexual partners [12]. In Germany, MSM represented 45.6% of users on online dating and social networking platforms [13]. In the USA, Rosenbaum et al. demonstrated that mobile social apps can facilitate the spread of syphilis and gonorrhea [14]. In South China, Guo reported that 63.8% of MSM utilized social software to seek sexual partners, emphasizing the geosocial app's significance in MSM communities [15]. Song's study showed that 53.7% of MSM reported having used gay apps to find sexual partners [11]. Hong et al. reported an HIV prevalence of 7.9% among gay app users and demonstrated that HIV infection was significantly associated with app use [16]. A behavior associated with higher-risk sexual practices and potential HIV infections, attributed to the rapid growth of internet technology [17].

The Blued, a prominent MSM mobile geosocial network App in China [18], plays a significant role in promoting healthy relationships among gay individuals and in advancing HIV prevention strategies, including the implementation of “Internet + HIV” models [19]. Internet and mobile apps revolutionize MSM dating, introducing discreet platforms [20,21]. Notably, Blued is widely used in the medical research, driving the optimized development of medical research and public health strategies [22,23]. According to a survey results, 98.2% of MSM in China have used gay apps at some point in their lives, with Blued being the most popular (97.5% usage rate). This is far higher than the usage of other platforms like Finka (18.4%), making Blued about 5.3 times more prevalent than other single platforms [24]. However, the lack of comprehensive MSM data in China presents significant challenges to the development and implementation of effective STD prevention strategies. The utilization of big data derived from social media platforms may offer a solution to this challenge, as technological advancements transform the socialization and partner-seeking behaviors of MSM.

Hainan Province, located in the southernmost region of China, is a key tourism and population hub near Southeast Asia. Between 2011 and 2019, young students in Hainan Province accounted for 5.99% of the annual HIV/AIDS cases. Notably, 91.38% of HIV-positive male students in higher education institutions acquired HIV through homosexual contact [25]. This trend indicated a potential uncontrolled transmission of HIV among college MSM students in Hainan. Accurately estimating MSM population within colleges is essential for calculating STD incidence in this group. However, the scarcity of comprehensive studies on college MSM in Hainan Province limits understanding and addressing the STD prevalence. To tackle this, we assessed the risks by estimating MSM population, distribution, and behaviors through Blued App to improve disease control.

## Methods

### Study colleges and universities

In Hainan Province, a total of 21 colleges and universities with 25 campuses, are enrolling students in 2023. These higher education institutions include 8 undergraduate colleges and 13 junior colleges, with disciplines spanning financial colleges (five), science and engineering colleges (four), comprehensive universities (four), medicine colleges (three), normal colleges (two), language and literature colleges (one), political science and law colleges (one), and sports colleges (one). The geographical distribution of the 25 campuses is as follows: 15 are located in Haikou City, six in Sanya City, and one each in Danzhou, Wuzhishan, Qionghai, and Wenchang City.

### The geographical characteristics and MSM population density analysis

We performed a comprehensive analysis of the geographical characteristics associated with the estimated population data of MSM across all colleges in Hainan Province. Initially, the total number of college users of the Blued App within the same City was calculated. The MSM population density across colleges in various regions of Hainan Province was determined by dividing the total MSM population by the number of male students enrolled in all colleges within the region. The MSM population density in colleges across different cities in Hainan Province was accurately mapped using ArcMap 10.8.1 to identify geographical hotspots.

### Exploratory analysis

Multiplier method is perhaps the most common because of its simplicity and ease of use [26,27]. The basic principle of this method is that the count of individuals from the target population observed at designated institutions within a defined time period is equal to the total size of the estimated population multiplied by the proportion of the population who attended the selected institutions. The multiplier formulas to estimate the size of MSM are following (1,2), where *N* is the estimated size of student MSM, *r* is the number of recorded individuals; *m* is the multiplier, *p* is the proportion of recorded individuals among observed population. *CI* represents the confidence interval of population size.


$$\begin{array}{c} N=r\times m=r\times 1/p \end{array}$$ (1)

$$\begin{array}{c} CI=N\pm z_{\alpha /2}\times r\times \sqrt{(\text{1}-p)/p^{\text{3}}\times n} \end{array}$$ (2)

In this study, *r* represents the number of MSM registered on Blued, and *p* is the proportion of student MSM who use the Blued App, as obtained from the questionnaire survey. Since the total number of MSM among college students is unknown, we consider that and *N* are approximately equal *n* in this study, and thus *N* is used as a substitute for *n* to calculate the *CI*. The average number of registered on these dates was used as the observed individuals, and the Multiplier method was employed to estimate MSM.

### Data collection and management

This study employed Blued App to examine MSM students across all colleges and universities in Hainan Province. Data collection from the Blued App was conducted in accordance with Blued’s Terms of Service and Privacy Policy.

In 2023, we collected data on Blued online users across colleges and universities in Hainan over ten consecutive days from May 9 to May 18 between 10:30–11:30 every night. Briefly, the Blued App was employed to locate the central location of the target campus and identify the radius according to the size of the campus (ranging from 0.20 to 0.90 km). Then, information on each user, encompassing nickname, age, height, weight, gender role, and online status was recorded. Users registered on the Blued platform via mobile phone verification and were identified by self-selected pseudonyms. This study utilized fully anonymized and aggregated data sourced exclusively from the platform's public domain. No personally identifiable information (PII) was collected or accessed. Furthermore, all datasets underwent a rigorous de-identification process prior to analysis to ensure individual privacy was maintained. At the same time, we also gathered pertinent data regarding student numbers from the Hainan Statistical Yearbook 2022.

Initially, data were gathered from individual colleges and subsequently integrated into a comprehensive dataset. This dataset was then meticulously examined for potential errors or discrepancies. For quality control, data from 3128 MSM aged 18–24 were analyzed, excluding 341 individuals due to anomalies like prolonged inactivity, age discrepancies, being outside the capture range, and inaccurate personal information, to ensure the efficient processing of the survey data. To gain a deeper understanding of Blued App usage within the MSM population, a pertinent questionnaire survey was carried out from April 6th to 10th, 2025, and obtained written informed consent to assess the reliability of utilizing Blued App in this study.

### The definition of the relevant indicators

In this study, the following definitions were adopted for the key terms used in the study.

Registered user: A unique Blued account observed within the campus geofence at any time during the 10 day study period, after removing duplicate accounts, used for estimating the total MSM population among college students in Hainan.

Active user: A registered users who were online for five or more days were defined as active users.

Online activity: The total number of male students enrolled in colleges in the same city of Hainan is summed, and the number of active users in this city is then divided by this total, and the ratio obtained indicates the online activity within the city. Sensitivity analyses using thresholds of four and six days yielded similar patterns, supporting the robustness of this definition.

Online user: A registered user whose status was online at the time of data collection.

Offline user: A registered user whose status was offline at the time of data collection but still appeared in the user list within the geofence.

Secret user: A registered user with ‘private’ mode enabled on Blued, making them invisible for matching. Only 37 such users were identified (2.34% of registered users), so their impact on the results is minimal.

BMI: Body Mass Index.

### Statistical analysis

The dependent variable is a binary indicator of online activity status, where a value of 1 denotes online activity lasting five days or more, and a value of 0 signifies online activity lasting less than five days. The independent variables include age, BMI, college level, college location, college classification, and university campus. Multivariate binary logistic regression was used to examine factors associated with active user status. Adjusted odds ratios (aOR) with 95%CI are reported. All tests were two-sided with α = 0.05, using SPSS 27.0.

### Ethical approval and informed consent

This study was conducted in accordance with the ethical principles of the Declaration of Helsinki and received approval from the Ethical Committee of Hainan Medical University, Hainan, China (HYLL-2024–838). This study utilized completely anonymous aggregated data, without involving any specific personal privacy information. Moreover, all the data were strictly safeguarded.

## Results

### The usage of Blued App in the Hainan MSM population

In this study, we conducted a questionnaire survey involving 181 MSM residing in Hainan regarding the usage of the Blued App. Of the 181 participants, 164 individuals had utilized the Blued App (90.61%), with college students comprising 15.47% (28/181). Among the 28 student MSM, 25 (89.28%) students reported engaging with the Blued App. Of those who have utilized the app, 76.00% (19/25) have been doing so for a duration exceeding two years. Additionally, our research reveals that 76.00% (19/25) of these student MSM primarily use Blued for seeking sexual partners, while 56.00% (14/25) utilize it for social interaction (**Table 1**).

**Table 1:** A survey on the usage of social media in student MSM.

Variables		Proportion (%)
**Education**		
	Senior high school	14.28(4/28)
	Junior college	10.71(3/28)
	Bachelor's degree or above	75.00(21/28)
**Dating apps (Multiple choice)**		
	Blued	89.28(25/28)
	Finka	50.00(14/28)
	Tiktok private message	42.85(12/28)
	Little Red Book private message	28.57(8/28)
**Duration of using Blued App**		
	≤6 months	20.00(5/25)
	7-12 months	12.00(3/25)
	2 years and above	76.00(19/25)
**The main purposes for using Blued (Multiple choice)**		
	Social communication and friends	56.00(14/25)
	Find sexual partners	76.00(19/25)
	Understand circle culture	8.00(2/25)
	No specific use	4.00(1/25)

### The number and behavior dynamics of college Blued users in Hainan Province

The data was collected from May 9th to 18th, 2023, over a period of ten consecutive days. The total number of online annotations was 3362. And 234 were eliminated for duplicate annotations and abnormal data, leaving 3128 as valid data. During the 10 day survey period, 2596 users had used the Blued App, and the average number of college students online on the Blued App was 939, while the total number of registered users was 1,579. Among these registered users, 2.34% (37/1,579) were identified as secret users (**Table 2**). According to the survey among MSM students, 89.28% reported being registered Blued (**Table 1)**. Thus, the estimated MSM size in colleges across Hainan Province was 3504 (95%CI: 3464–3544).

**Table 2:** The number of MSM using the Blued App in colleges on Hainan Island, China (n).

Users status	Dates in 2023										Average
	9 May	10 May	11 May	12 May	13 May	14 May	15 May	16 May	17 May	18 May	
Online	897	900	937	913	905	959	955	992	993	941	939
Offline	540	525	613	583	601	595	608	624	665	683	604
Secret	34	34	40	34	35	33	44	37	38	36	37
Registrants	1471	1459	1590	1530	1541	1587	1607	1653	1696	1660	1579

### The influencing factors of online activity of the MSM population at Hainan colleges and universities

We found that the proportion of active users was 33.13% (860/2596), and the proportion has statistically significant associations (*P* < 0.05) between age, college level, city, and various colleges, indicating their pivotal roles in shaping the online activity patterns of MSM in Hainan colleges. N represents the proportion of users who engaged with the Blued software online for at least five days within the total caseload. Notably, Danzhou and Wenchang, relatively small urban areas with limited Blued registrants, exhibited a high proportion of active users (42.22% and 41.30%). The multivariate logistic regression analysis identified that users aged 18−19 years (aOR = 1.49, 95%CI: 0.20–1.84) and 20−21 years (aOR=1.52, 95%CI: 1.26–1.85) exhibited a higher likelihood of being active users than individuals aged 22−24 years. Additionally, the proportion of active users among MSM from undergraduate colleges was 1.90 times (95%CI: 1.38–2.61) greater than that among those from junior colleges. In terms of the colleges’ category, the normal colleges (aOR = 0.55, 95%CI: 0.36−0.84) and the sports colleges (aOR = 0.27, 95%CI: 0.10−0.72) had a higher proportion of active users than comprehensive universities. (**Table 3**)

**Table 3:** The determinants on the Blued App usage days of the student MSM in Hainan colleges, China. (n = 2596).

Variables		Total	Active users, N^a^ (%)	Multivariate logistic regression	
				aOR (95%CI)	P
Age, years					
	18-19	637	36.58(233/637)	1.56(1.27-1.92)	<0.001
	20-21	858	38.11(327/858)	1.56(1.29-1.89)	<0.001
	22-24	1101	27.25(300/1101)	Ref.	
BMI					
	Thinnish	465	36.56(170/465)	0.86(0.51-1.44)	0.556
	Normal	1879	32.57(612/1879)	0.74(0.45-1.21)	0.225
	Overweight	187	28.34(53/187)	0.58(0.33-1.04)	0.067
	Obesity	65	38.46(25/65)	Ref.	
Colleges level					
	Undergraduate colleges	1947	34.98(681/1947)	1.81(1.41-2.32)	<0.001
	Junior colleges	649	27.58(179/649)	Ref.	
City					
	Haikou	1868	32.28(603/1868)	2.30(0.67-7.88)	0.183
	Sanya	532	37.03(197/532)	4.14(1.56-10.98)	0.004
	Wenchang	46	41.30(19/46)	4.25(1.02-17.61)	0.046
	Danzhou	45	42.22(19/45)	2.56(0.64-10.07)	0.183
	Qionghai	67	25.37(17/67)	4.18(1.35-12.93)	0.013
	Wuzhishan	38	13.16(5/38)	Ref.	
Colleges category					
	Financial colleges	399	41.10(164/399)	1.00(0.58-1.73)	0.990
	Science and engineering colleges	390	31.79(124/390)	0.57(0.25-1.30)	0.184
	Normal colleges	527	26.94(142/527)	0.55(0.36-0.84)	0.005
	Sports colleges	55	9.09(5/55)	0.27(0.10-0.72)	0.009
	Medical colleges	237	31.22(74/237)	0.82(0.57-1.19)	0.301
	Language and Literature colleges	46	41.30(19/46)	–	–
	Political science and law colleges	59	33.90(20/59)	1.55(0.82-2.94)	0.174
	Comprehensive universities	883	35.33(312/883)	Ref.	
Colleges					
	Haikou A1	377	31.56(119/377)	0.74(0.53-1.03)	0.074
	Haikou D	253	47.04(119/253)	1.17(0.66-2.07)	0.589
	Sanya B	208	38.46(80/208)	0.47(0.20-1.08)	0.073
	Haikou B1	191	28.80(55/191)	1.14(0.72-1.78)	0.578
	Haikou E	176	22.16(39/176)	0.76(0.47-1.22)	0.257
	Others	1391	32.21(448/1391)	Ref.	
Total		2596	33.13(860/2596)		

N^a^ represents the proportion of users who engaged with the Blued software online for at least five days within the total caseload.

### Geographical distribution of MSM population in colleges and universities in Hainan Province

**Fig 1:**
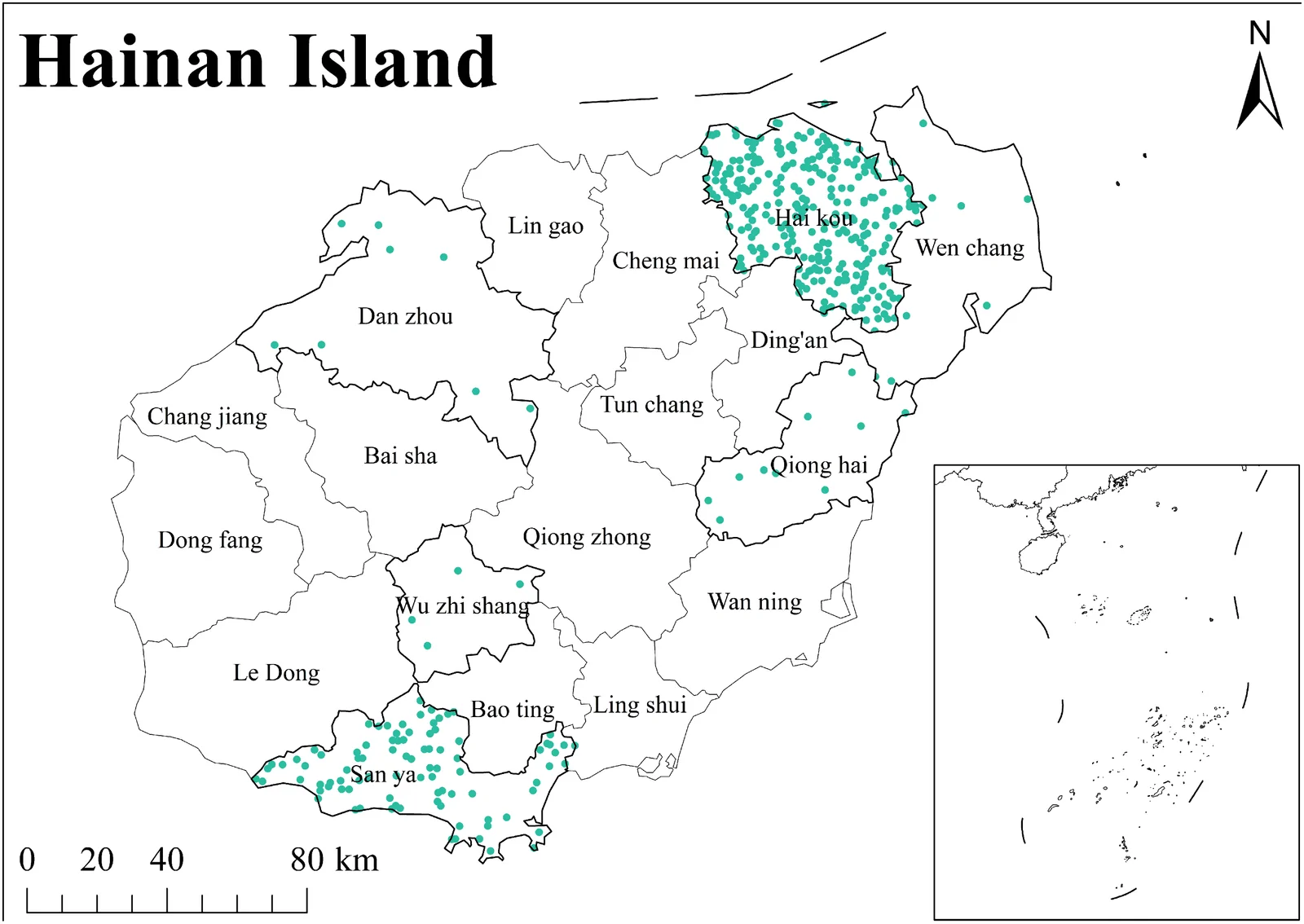
The geographical distributions of student MSM in Hainan Province. The blue dots in Fig 1 represent the estimated geographic densities of MSM populations in colleges across different regions of Hainan. The denser he blue dots are, the higher the concentration of MSM populations in that region, while areas with fewer dots indicate lower concentrations. Map source: National Standard Map Service Website of the Ministry of Natural Resources of the People's Republic of China (http://bzdt.ch.mnr.gov.cn/), Approval No. GS (2024) 0650.

**Fig 2:**
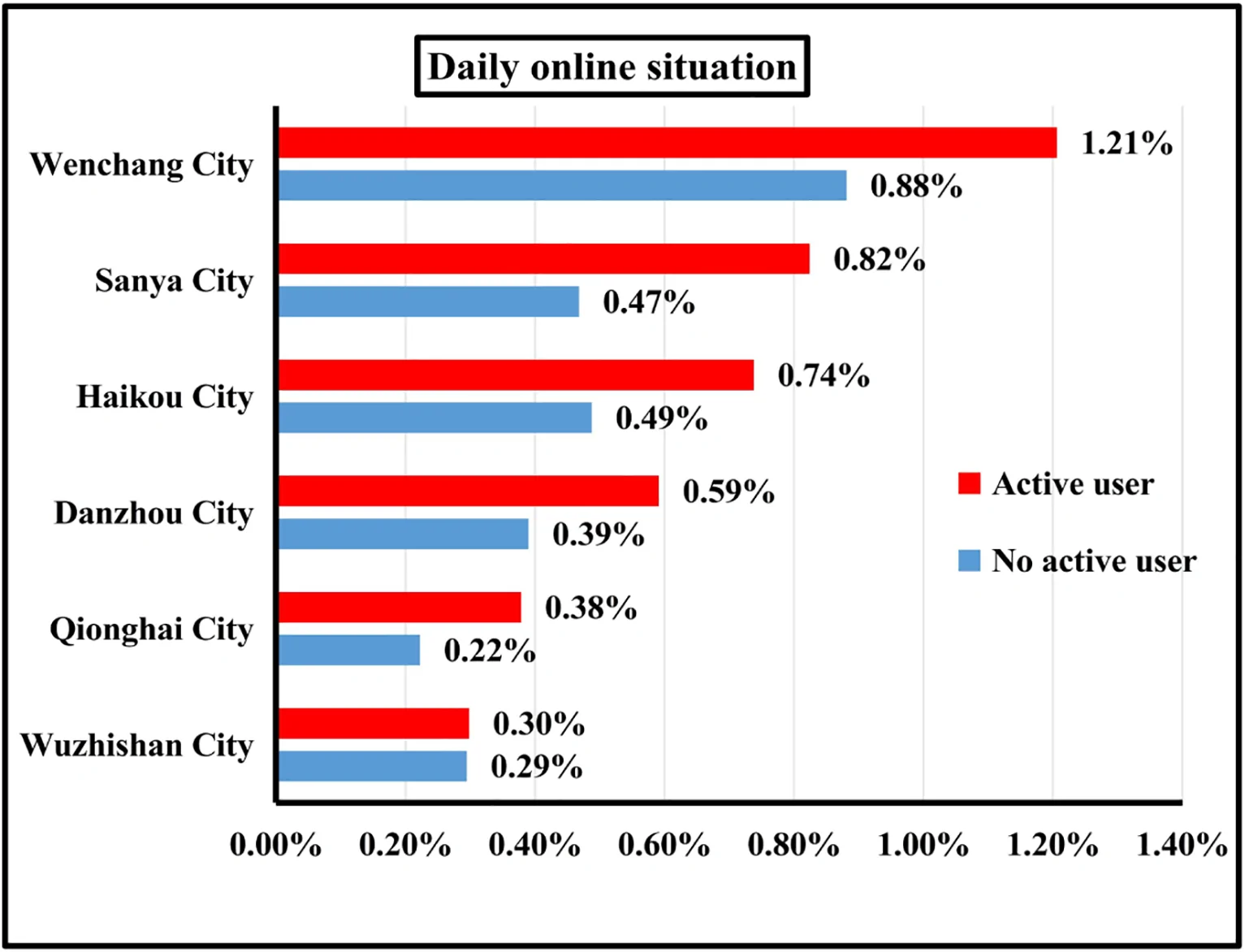
The distribution of active users in cities and counties of colleges across Hainan Province.

The results showed that the MSM population in colleges and universities in Hainan Province was mainly concentrated in Haikou, accounting for 72.22% (2259/3128), while Sanya ranked second with 20.75% (649/3128). The distribution of other cities was relatively scattered, with no notable patterns of aggregation established (Fig 1). We also found that the highest online activity was in Wenchang at 1.21% (19/1600), followed by Sanya at 0.82% (208/25169), and Haikou at 0.74% (663/89827) (Fig 2).

## Discussion

The MSM population exhibits a higher risk for STD than the general population, including HIV, syphilis, hepatitis B virus, and other infections. Student MSM has also become a high-risk population for STD in China [2]. Understanding the size and distribution of the MSM population is crucial for effectively preventing AIDS and achieving the UNAIDS 95-95-95 targets by 2030 [28]. The significance of this study lies in providing data to support more effective prevention and control of the spread of STD among MSM by accurately estimating the size and distribution of the MSM population among male college students in Hainan.

We utilized the multiplier method in conjunction with the Blued weekend usage rate, as determined by a questionnaire survey, to estimate the size of the college student MSM across Hainan Province. The decision to focus on weekend usage rates was made because the extended duration of the workweek does not accurately represent daily usage patterns. The sample values for all universities fall within their respective 95% confidence intervals. The influence of the multiplier adjustment coefficient (0.893) on the overall population is negligible. Finally, we estimated the overall size of college student MSM cross Hainan Province was 3504.

At present, similar studies in China are relatively scarce, and they only focus on a few cities with relatively high social and economic status. This study focuses on a descriptive analysis of Blued-registered MSM in Hainan colleges, including geographic distribution and user characteristics. In Hainan, the Haikou City and Sanya City were identified as hotspots. The primary factor contributing to Haikou and Sanya is the concentration of universities in Hainan Province within these two cities. A comprehensive analysis suggests that this phenomenon may be associated with the regional level of economic development. Furthermore, economically developed metropolitan exhibit more active MSM communities, attributable to their superior network infrastructure and high levels of Internet penetration. This pattern is consistent with the geographic distribution characteristics of the MSM population in mainland China, where economically developed regions tend to have a more dispersed MSM population [29]. Thus, the economic development level might be an important factor in the distribution of social networking MSM.

The current study revealed that the proportion of Blued App users among male college students ranged from 0.44% to 6.67%, which is lower than the 3.7% to 8.5% reported in a previous study [30]. Our results indicated that a significant proportion of MSM students in Hainan colleges actively utilize the Blued App, predominantly for social interaction and seeking sexual partners. These results aligned with previous studies conducted in China [11]. In modern society, digital communication technologies, encompassing social media platforms and the Internet, have emerged as the predominant channels for MSM to facilitate social interactions and seek partners. These platforms expedite the establishment of social connections and the identification of sexual partners, potentially increasing the risk of HIV transmission [31,32]. Furthermore, these tools offer significant potential for identifying high-risk populations and improving risk surveillance.

Prior studies have demonstrated that the strategic use of social media can substantially increase the detection rate of HIV infections among MSM in Chinese colleges, thereby reducing sexual risk behaviors [22]. Moreover, a comprehensive management framework delivered via the Internet, which integrates HIV prevention, treatment, and associated technologies, has proven effective in reducing HIV incidence among MSM populations [33–35]. Previous research has highlighted the importance of software such as Blued in conjunction with Internet data sources to provide and deliver health knowledge. For example, this model could be used to push information about the correct use of condoms to people with suspicious high-risk behavior labels [36]. Additionally, considering that MSM frequently encounters discrimination and marginalization within social networks—obstacles that impede access to HIV and syphilis testing and treatment—leveraging the influence of key individuals within these networks may serve as an effective strategy to mitigate stigma and prejudice [37].

This study employed the multiplicative method to preliminarily explore the size of the MSM among college students. First, a potential coverage bias exists in the estimation of the MSM population size. The multiplier method relies on the proportion of MSM who use Blued (89.28%), which was derived from a convenience sample of 28 college students who self-identified as MSM. This sample may overrepresent gay-identified students who are active on geosocial apps, while underrepresenting bisexual male students, MSM but do not identify as gay, and those who do not use any dedicated gay apps. Consequently, the estimated coverage rate is likely an overestimate, and the total MSM population size derived from the multiplier method is likely an underestimate of the true MSM population. Therefore, our results should be interpreted as the lower bound of the MSM population among Hainan college students, or more conservatively, as the Blued-observable MSM population rather than the total MSM population

Furthermore, the inherent mobility of MSM individuals during the data collection period may violate this assumption, representing a limitation. To address this, we implemented three key strategies: (1) the college student population is relatively stable, (2) the surveys exclusively were conducted on weekdays to minimize transient population effects, and (3) the data collection was completed within a condensed timeframe to reduce exposure to migration influences. Additionally, while Blued App is a widely used platform among MSM in Chinese colleges, not all MSM individuals use this platform, potentially leading to an underestimation of the MSM population size. Lastly, some MSM individuals maintain multiple Blued App accounts simultaneously, which could result in double counting and consequently overestimate the size of the MSM population.

## Conclusions

Blued App is widely used among student MSM in Hainan Province, especially for seeking sexual partners. Although Haikou and Sanya are the main hotspots, smaller cities like Danzhou and Wenchang show disproportionately high active user proportions. These results highlight the need for targeted sexual health education and STD surveillance in specific colleges, including those in less populous cities with high online sexual networking activity.

## Supporting information

S1 File S1 Data. The dataset of this article.
(XLSX)
